# Albumin-Based Cryogels as Floating Platforms for Gastroretentive Drug Delivery Applications

**DOI:** 10.1021/acsomega.5c04153

**Published:** 2025-08-13

**Authors:** Wei-Chin Hsu, Teh-Min Hu

**Affiliations:** Department of Pharmacy, College of Pharmaceutical Sciences, 34882 National Yang Ming Chiao Tung University, Taipei 112304, Taiwan

## Abstract

Gastroretentive drug delivery systems (GRDDS) are attractive oral extended-release dosage forms that prolong drug release and absorption in the gastrointestinal tract through engineered mechanisms to extend the residence time of orally administered dosage forms in the stomach. One of the gastroretentive designs is to render the dosage forms floatable in the gastric fluid upon oral administration. The present study aimed to develop albumin cryogels with extended buoyancy and remarkable resistance to gastric proteolysis. Protein hydrogel monoliths can be prepared through thiol-organosilane-mediated hydrogelation of bovine serum albumin, followed by lyophilization to remove water, thereby producing cryogels. The study demonstrates that, by adjusting the composition of Na_2_HPO_4_ and sodium alginate in the hydrogel formulation, protein degradability can be effectively tuned to produce an intact floating structure with sustained buoyancy for over 24 h in simulated gastric fluid. This result is consistent with the finding that fluorescein, as a model payload, generally exhibited a decreased release rate from the formulations with increasing concentrations of Na_2_HPO_4_ and alginate. Notably, above a threshold concentration (i.e., about 2%) of alginate, the hydrogel monolith rapidly disintegrated in nearly neutral environments such as water and simulated intestinal fluid, exhibiting pH-responsive characteristics. Finally, the generalizability of the albumin–organosilane–alginate system as a promising controlled-release platform is further demonstrated in expanded application examples, using doxorubicin, mitoxantrone, methylene blue, and rhodamine 6G as the loaded payload; all exhibiting reproducible controlled-release profiles for the alginate-doped formulation. This finding can be explained by the SEM images showing smaller and denser porous structures with thicker, fiber-tangled intercompartment walls for gels with high alginate concentrations. Overall, the freeze-dried albumin–organosilane–alginate cryogel formulation demonstrates excellent buoyancy, resistance to degradation by simulated gastric fluid, and the ability to regulate drug release, highlighting its feasibility as a GRDDS.

## Graphical abstract

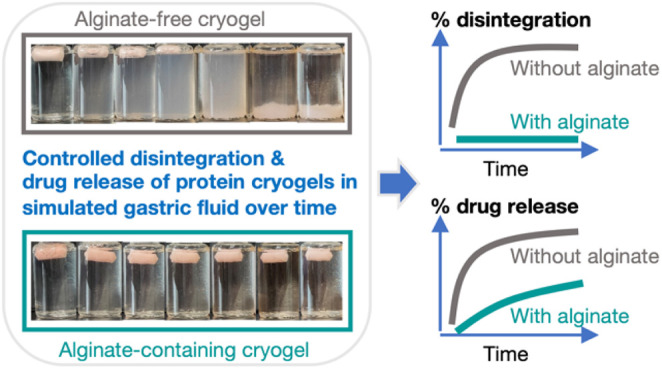

## 1 Introduction

Oral administration is the most common and preferred route for systemic drug delivery due to its convenience, safety, and patient compliance. However, drugs targeting the upper gastrointestinal (GI) tract face several challenges, including rapid gastric emptying, enzymatic degradation, poor solubility at intestinal pH, and narrow absorption windows.^1^ These limitations can lead to suboptimal drug absorption and reduced therapeutic efficacy. Gastroretentive drug delivery systems (GRDDS) have been developed to address these issues by prolonging the residence time of dosage forms in the stomach, allowing extended drug release and improved bioavailability.^2–4^

Several GRDDS strategies have been proposed, including floating systems, mucoadhesive systems, expandable or swellable matrices, high-density systems, and magnetic retention systems.^5,6^ Among these, floating systems are the most widely investigated and clinically promising due to their passive mechanism of gastric retention and relatively simple formulation requirements.^7^ Floating systems maintain buoyancy in gastric fluid, allowing prolonged contact with the gastric mucosa, which is particularly advantageous for drugs with narrow absorption windows (e.g., levodopa, ciprofloxacin), local gastric action (e.g., antacids, antibiotics for *H. pylori*), or poor intestinal stability.^5–11^

Cryogels, macroporous networks fabricated under subzero conditions and dried via lyophilization, have gained attention as versatile biomaterials for drug delivery and tissue engineering.^12–15^ Particularly, biobased cryogels derived from natural polymers such as gelatin, alginate, chitosan, and cellulose offer attractive biocompatibility, biodegradability, and structural flexibility.^16–21^ Despite extensive exploration of cryogels for injectable scaffolds and controlled-release implants,^22^ their application as floating gastroretentive drug delivery platforms remains largely underexplored. In this study, we developed and optimized an albumin-based cryogel to address this gap, aiming to combine buoyancy, gastric stability, and tunable drug release.

Albumin offers a unique set of advantages that make it an attractive material for biomedical and pharmaceutical applications.^23^ As a naturally abundant plasma protein, albumin is inherently biocompatible and biodegradable. Structurally, albumin contains multiple binding sites for a wide variety of drugs, particularly hydrophobic compounds, enabling it to function as an effective drug carrier with potential to improve solubility, stability, and pharmacokinetics.^24,25^ In addition to its pharmaceutical versatility, albumin can be easily modified chemically to tailor its mechanical, degradation, or release properties.^26,27^ Beyond its biological functionality, albumin is a renewable, bioresourced material that can be sustainably sourced from agricultural and clinical byproducts.^28^ However, oral drug delivery using albumin or other proteins as the main component of the delivery vehicle may seem counterintuitive, as proteins typically undergo extensive degradation mediated by gastric enzymes.

In our previous work, we developed a protein-based hydrogel composed of bovine serum albumin and a thiol-functionalized organosilane, formed via a mild, one-pot gelation process under neutral pH.^29,30^ We previously demonstrated that the hydrogel structure consists of chemically cross-linked polycondensed siloxane species (based on solid-state ^29^Si NMR) and is physically integrated with partially denatured protein structures, as evidenced by FTIR and fluorescence spectroscopy.^29,30^ Following lyophilization, this hydrogel transforms into a porous cryogel with excellent buoyancy—an essential trait for floating GRDDS. However, a key limitation emerged: the protein matrix, while biodegradable and biocompatible, is highly susceptible to degradation by pepsin in gastric fluid, leading to premature disintegration.

To overcome the challenge of proteolytic degradation, the present study focuses on enhancing the structural and enzymatic stability of albumin-based floating cryogels—while preserving their buoyancy and capacity for controlled drug release. We hypothesized that reinforcing the cryogel matrix with sodium phosphate and incorporating sodium alginate, a naturally derived polysaccharide known to inhibit pepsin activity,^31,32^ would improve the cryogel’s gastric resilience. A combination of drug release studies and microstructural analysis was used to evaluate the effects of these formulation strategies. Through this approach, we aim to establish an albumin-based, biocompatible cryogel platform suitable for gastroretentive drug delivery and adaptable to broader applications, including localized gastric therapies, probiotic delivery, and diagnostic technologies.

## 2 Materials and Methods

### 2.1 Materials

The following materials were purchased from Sigma-Aldrich: 3-mercaptopropyltrimethoxysilane (≥95%), (3-mercaptopropyl) methyldimethoxysilane (≥95.0%), bovine serum albumin (heat shock fraction, 98%), sodium chloride, sodium fluorescein, rhodamine 6G hydrochloride, methylene blue, doxorubicin hydrochloride, pepsin (≥400 units/mg protein), sodium alginate, and sodium hydroxide. The following materials were obtained from J.T. Baker: potassium phosphate, monobasic (KH_2_PO_4_), sodium phosphate, dibasic (Na_2_HPO_4_), potassium chloride, and hydrochloric acid. Mitoxantrone hydrochloride was purchased from Cayman Chemical. All chemicals were used as received. Deionized water was used throughout the experiments.

### 2.2 Preparation of Lyophilized Hydrogels

Unless otherwise mentioned, the basic composition of the hydrogel included albumin, 3-mercaptopropyltrimethoxysilane (MPTMS), Na_2_HPO_4_, and water, with or without sodium alginate. Concentrations of each substance were varied in individual experiments and will be elaborated in the following sections. After mixing the above chemicals in a 1 mL aqueous solution in a 2 mL Eppendorf tube, the resulting emulsion was left standing at room temperature (about 23 °C) for 24 h or at 50 °C for 1 h to form the hydrogel. It was then prefrozen at −80 °C overnight, followed by freeze-drying overnight using a lyophilizer (VirTis Co., NY, USA). The final freeze-dried product, with the shape of its container, could be easily removed from the Eppendorf tube.

### 2.3 Degradability Tests

The factors affecting the physical stability of protein cryogels in the simulated gastric fluid were investigated by varying the gel compositions. The preparation of the simulated gastric fluid (SGF), according to the United States Pharmacopoeia (USP), contained 2 g NaCl, 3.2 g pepsin, and 7 mL HCl in 1000 mL water, pH 1.2. First, the monolithic cryogels and the vials containing SGF or water were separately weighed. Subsequently, the cryogels were placed into the SGF-containing vials. At the predetermined time points, the undegraded solid structures, when they remained floatable and retrievable, were removed from the fluid surface. After waiting for the absorbed fluid to drain, without squeezing or exerting pressure, back into the vial, the gel structure was put aside and the fluid-containing vial was directly weighed. The gel degradability (%) was quantified by comparing the fluid absorption ratio (*F*) of the cryogels in SGF to those in water. It was calculated as

$$\mathrm{Fluid absorption ratio}:F=\left(W_{0}-W_{t}\right)/W_{\mathrm{gel}}$$

$$\mathrm{Degradability}\left(\%\right)=\left[\left(F_{\mathrm{water}}-F_{\mathrm{SGF}}\right)/F_{\mathrm{water}}\right]\times 100\%$$

Where *W*_0_ represents the initial weight of the vial containing SGF or water. *W*_t_ represents the weight of the vial at the predetermined time point, *W*_gel_ represents the initial weight of the dry cryogel. *F*_water_ represents the fluid absorption ratio of the cryogel placed in water, *F*_SGF_ represents the fluid absorption ratio of the cryogel placed in SGF.

### 2.4 Loading and Release of Model Compounds

The cryogels were prepared at a final concentration of 100 μM of various model compounds in aqueous solutions containing 5% w/v albumin, 120 mM MPTMS, 240 mM Na_2_HPO_4_, and 2% w/v alginate, unless otherwise stated. The model payloads included fluorescein, methylene blue, doxorubicin, rhodamine 6G, and mitoxantrone. These compounds were chosen mainly because of their fluorescent properties, allowing fast determinations. Besides, the chosen molecules exhibit certain similarities and dissimilarities in their molecular structures and physicochemical properties. Following the gelation and freeze-drying processes, the cryogels were placed in 20 mL of simulated gastric fluid (SGF) at room temperature while being agitated at 100 rpm using an orbital shaker. The room temperature protocol was adopted according to a pretest, showing insignificant difference in the release profile between room temperature and 37 °C. At the predetermined time points, 150 μL of the release medium was withdrawn (followed by adding back an equal volume of SGF to maintain constant volume) and centrifuged at 9400*g* for 10 min at 23 °C. After centrifugation, the supernatant was analyzed by fluorometric measurements for drugs released. The excitation/emission wavelengths of fluorescein, methylene blue, doxorubicin, rhodamine 6G, and mitoxantrone were 460 nm/523 nm, 610 nm/690 nm, 479 nm/593 nm, 480 nm/556 nm, and 610 nm/685 nm, respectively.

### 2.5 Mathematical Model Fitting of the Release Profile

The measured release profiles were fitted to various mathematical models of drug release using DDsolver, an add-in program in Microsoft Excel.^33^ It employs the nonlinear least-squares curve-fitting technique to fit kinetic drug-release models to release profile data sets. The average cumulative percentage of drug release (*n* = 3) was entered into an Excel spreadsheet according to the program’s required format. Subsequently, the following kinetic drug-release equations,^34^ built into DDsolver by the developers, were applied to the data sets.

$$\mathrm{Zero‐order}:f=k_{0}\times t$$

$$\mathrm{First‐order}:f=f_{\mathrm{Max}}\times \left[1-e^{\left(-k_{1}\times t\right)}\right]$$

$$\mathrm{Higuchi}:f=k_{H}\times \left(t^{0.5}\right)$$

$$\mathrm{Hixson‐Crowell}:f=100\times \left[1-{\left(1-k_{HC}\times t\right)}^{3}\right]$$

$$\mathrm{Korsmeyer‐Peppas}:f=k_{KP}\times \left(t^{n}\right)$$

Where *f* represents the percentage of drug released at time *t*. *f*_Max_ represents the maximum percentage of drug released. The parameters, *k*_0_, *k*_1_, *k*_H_, *k*_HC_, and *k*_KP_, denote the release rate constants associated with the zero-order, first-order, Higuchi, Hixson-Crowell, and Korsmeyer-Peppas equations, respectively. The exponent *n* of the Korsmeyer-Peppas equation is a parameter used for discerning drug-release mechanisms.

The goodness-of-fit of the different models was accessed through Akaike Information Criterion (AIC) and the adjusted coefficient of determination (*R* ^2^_adjusted_) provided by the program after modeling. The best-fitted model is the one with the lowest AIC values and the highest *R* ^2^_adjusted_ values.

### 2.6 pH Responsiveness

The cryogel monoliths with varying concentrations of sodium alginate (1–4%) were prepared, with other gel compositions fixed at 5% w/v BSA, 120 mM MPTMS, and 240 mM Na_2_HPO_4_. The simulated intestinal fluid (SIF) was prepared by dissolving 0.6805 g KH_2_PO_4_ in 100 mL water and adjusting pH to 6.8 with 0.2 M NaOH. The pH-dependent physical stability of the as-prepared cryogels was investigated in two experiments. First, the cryogels were placed in 10 mL of water, SIF, or SGF to monitor their structural integrity over time. Second, the pH-responsive disintegration of cryogels was demonstrated by first placing the cryogel in 10 mL of SGF for 1 h, followed by adding KH_2_PO_4_ (200 mM) and NaOH (0.2 N) to adjust the pH value to 6.8. The monolithic structure was examined in both the acidic and basic phases.

### 2.7 SEM Images

The cryogels were prepared from 5% w/v albumin, 120 mM MPTMS, 240 mM Na_2_HPO_4_, and 0–2% w/v alginate. The gel samples were platinum-coated (10 mA, 60 s using an Auto Fine Coater, JFC-1600, JEOL, Japan) to improve electrical conductivity. SEM images were captured using a JSM-7600F field emission scanning electron microscope (JEOL, Japan) operating at 5 kV incident energy.

## 3 Results and Discussion

### 3.1 Controlled Degradability of Albumin Cryogels in Simulated Gastric Fluid

To prepare albumin cryogels that are resistant to gastric fluid-mediated degradation, several formulation parameters were investigated, including variations in the gel composition of dibasic phosphate, sodium alginate, and organosilanes. Figure 1 shows decreased degradation with increased concentrations of Na_2_HPO_4_ in the gel.

**Figure 1:**
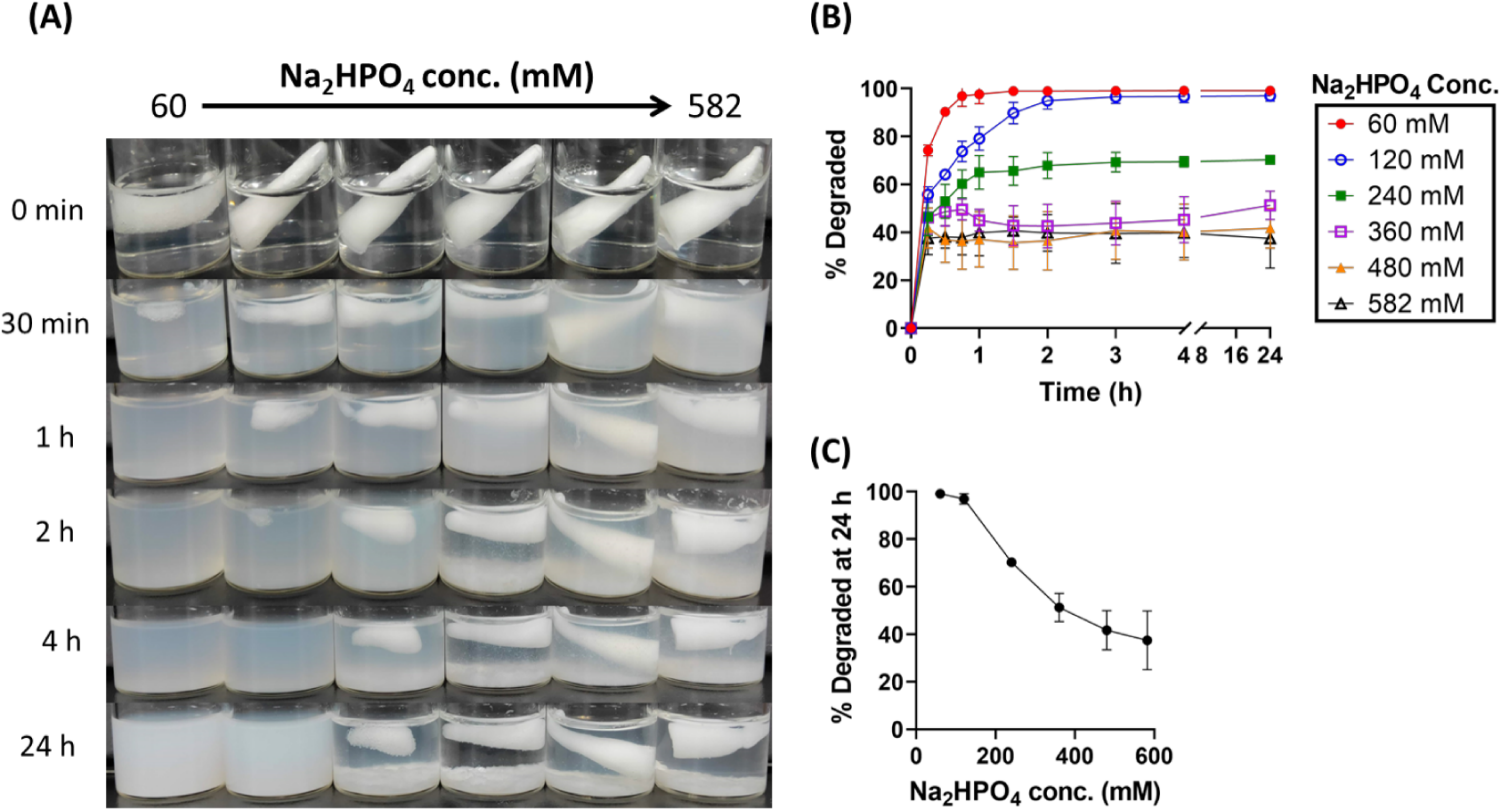
Degradability of albumin cryogels in simulated gastric fluid (SGF) as a function of Na_2_HPO_4_ concentration in the gel formulation. (A) Visual appearance of cryogels degraded in 5 mL SGF over time. (B) Time course of cryogel degradation. (C) Cryogel degradation percentage at 24 h plotted against Na_2_HPO_4_ concentration. Cryogel composition: 5% (w/v) bovine serum albumin (BSA), 60 mM MPTMS, 60 mM MPMDMS, and Na_2_HPO_4_ ranging from 60 to 582 mM.

The addition of sodium alginate, an inhibitor of pepsin,^31,32^ in the gel further reduced the gel degradation rate in a concentration-dependent manner (Figure 2A,B). Specifically, at 0.5% alginate, the degradation profile exhibits controlled degradation at a constant rate of 7.3% per hour over the period of 0.5–4 h (Figure 2A). Furthermore, at a fixed alginate concentration of 0.5%, increasing the concentration of dibasic phosphate in the formulation further decreased the initial degradation rate up to 4 h (Figure 2C); however, in the presence of alginate, the effect of phosphate seemed to be less obvious at 24 h (Figure 2C,D).

**Figure 2:**
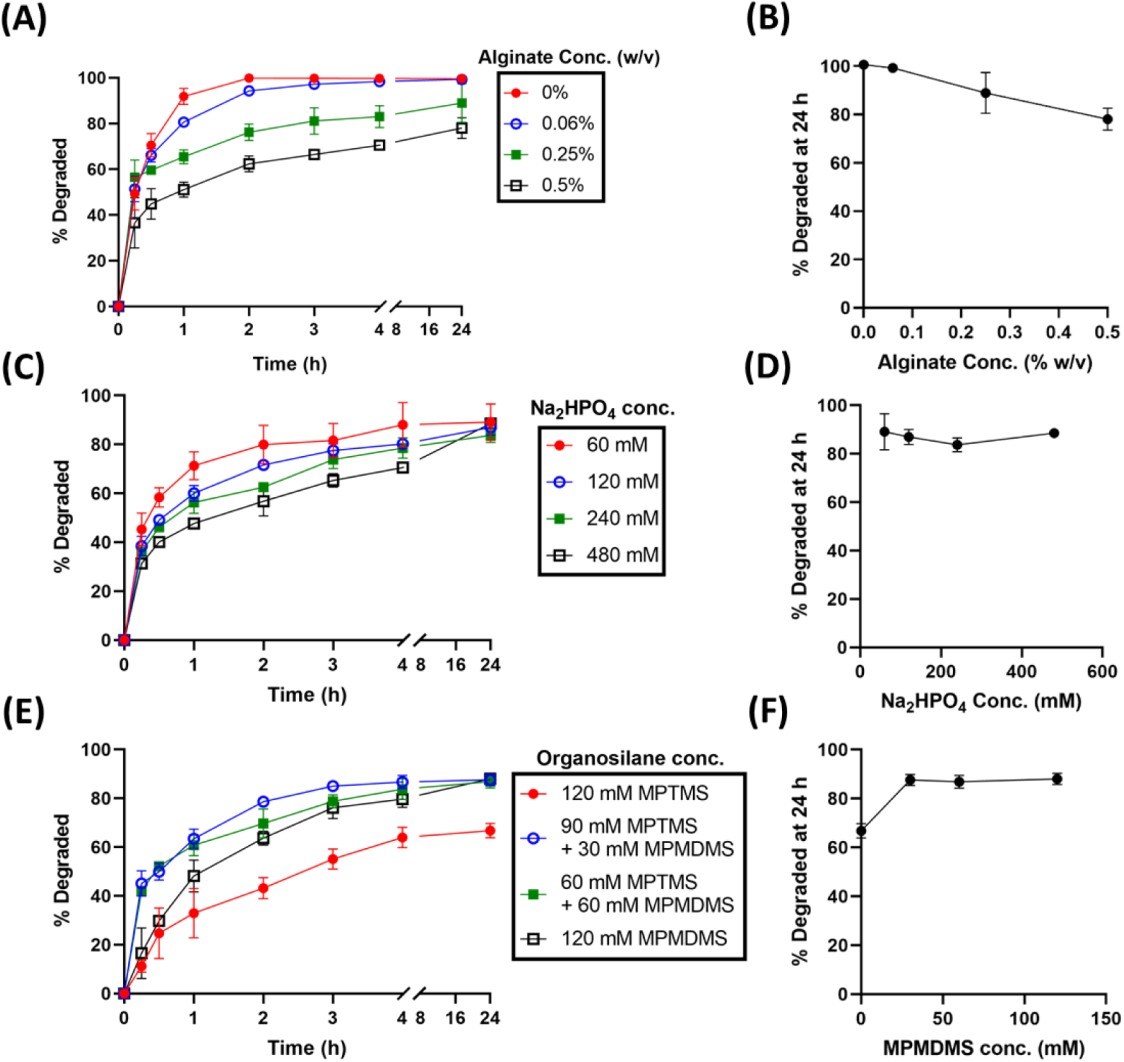
Controlled degradation of albumin cryogels in SGF by varying gel compositions. (A) Time course degradation profiles in 10 mL SGF with different alginate concentrations in gels. Other gel components were fixed at 5% (w/v) BSA, 60 mM MPTMS, 60 mM MPMDMS, and 120 mM Na_2_HPO_4_. (B) Cryogel degradation at 24 h vs alginate concentration in gels. (C) Degradation profiles for varying Na_2_HPO_4_ concentrations in gels. Other gel components were fixed at 5% (w/v) BSA, 60 mM MPTMS, 60 mM MPMDMS, 120 mM Na_2_HPO_4_, and 0.5% (w/v) alginate. (D) 24 h degradation vs Na_2_HPO_4_ concentration. (E, F) Effect of different organosilane compositions on gel degradation. Other gel components were fixed at 5% (w/v) BSA and 240 mM Na_2_HPO_4_. Panels show degradation profiles (E) and corresponding 24 h degradation (F).

The effect of organosilanes on gel degradability was evaluated by comparing single-silane and dual-silane systems at a total silane concentration of 120 mM. The results indicate that, compared with all other preparations, the albumin cryogel prepared with MPTMS alone exhibited better-controlled degradability (Figure 2E). The addition of (3-mercaptopropyl) methyldimethoxysilane (MPMDMS) did not offer formulation advantages in terms of controlled degradability (Figure 2F).

Thus, incorporating a second silane precursor into the gel formulation to create a heterogeneous siloxane network did not improve the cryogel’s degradation profile in simulated gastric fluid. This outcome may be attributed to differences in the chemical structure and cross-linking capacity of the two silanes. In our previous report,^30^ solid-state ^29^Si NMR analysis revealed that the mixed-silane system contains both D-type species (D^1^, D^2^) from MPMDMS and T-type species (T^2^, T^3^) from MPTMS. Because MPTMS contains three hydrolyzable methoxy groups, it can undergo more extensive siloxane condensation, leading to a denser, three-dimensional network. In contrast, MPMDMS, with only two methoxy groups, tends to form more linear or branched structures with lower cross-link density.

This structural difference was also supported by our previous SEM analysis.^30^ Cryogels prepared with MPTMS alone exhibited ordered lamellar morphologies with compact, continuous walls, while MPMDMS-containing gels showed more disordered, granular, and fibrous textures. Furthermore, the MPTMS-based system exhibited lower water absorption and higher surface hydrophobicity than the more hydrophilic MPMDMS-containing system.^30^ These findings suggest that the MPTMS-only formulation results in a more mechanically robust and hydrophobic cryogel matrix, which is less susceptible to enzymatic degradation in SGF.

### 3.2 Controlled Release of Fluorescein

To demonstrate the controlled-release capability of the albumin cryogel, we first used a fluorescent dye, sodium fluorescein, as a model payload. The basic cryogel formulation contained 5% w/v bovine serum albumin and 120 mM MPTMS, with varying concentrations of alginate and phosphate. Figure 3 shows the effect of changing alginate concentrations in the albumin cryogel (with [Na_2_HPO_4_] fixed at 240 mM) on the release profiles of fluorescein in the SGF. In the absence of alginate, complete release of fluorescein was observed within 2 h (Figure 3B). This finding aligned with the rapid degradation of the gel monolith (Figure 3B). When the albumin cryogel was formulated with added alginate, it significantly reduced the rate of fluorescein release with increasing concentrations of alginate (above 0.25%), corresponding with reduced degradation of cryogel structure in the SGF (Figure 3A,B). Interestingly, variable buoyancy was observed for cryogels formulated with alginate concentrations in the range of 0.25–1%. For example, the albumin cryogel prepared with 1% alginate exhibited an initial, short period of sinking before complete flotation at 1h (Figure 3A). A similar phenomenon was occasionally observed for preparations with lower alginate concentrations in the triplicate experiments. Nevertheless, the albumin gel prepared with 2% w/v alginate showed consistent and reproducible buoyancy in every repeated experiment. Based on this finding, 2% alginate was used as a fixed formulation parameter throughout the following experiments.

**Figure 3:**
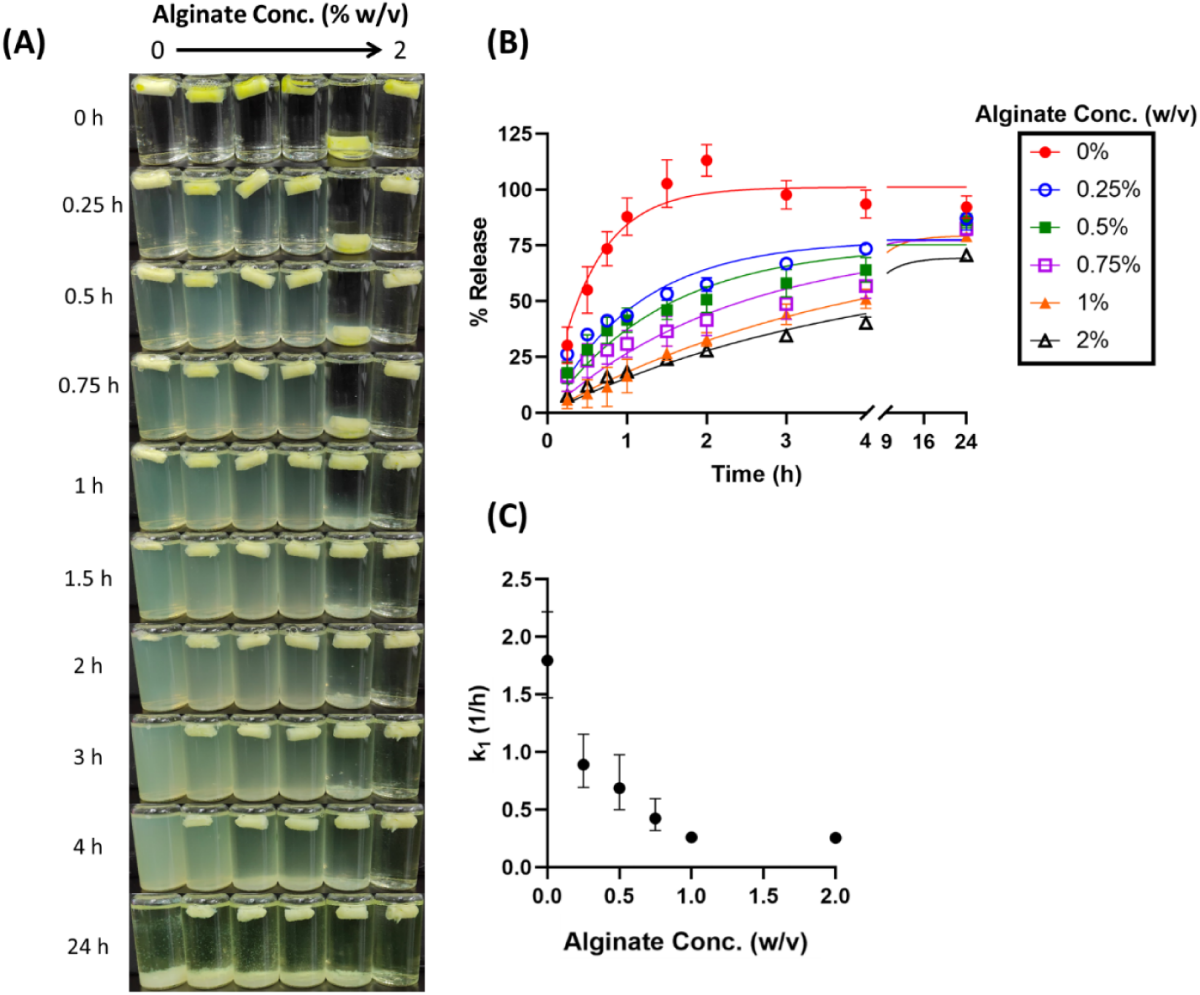
Effect of alginate concentration on fluorescein release in SGF. (A) Visual appearance of cryogels over time. (B) Cumulative release profile with observed data (dots) and first-order model fits (lines). (C) First-order release rate constants (*k*_1_) for each formulation.

To gain insight into the release mechanism, we analyzed the release profiles using various mathematical models. The goodness-of-fit measures, Akaike Information Criterion (AIC) and adjusted coefficient of determination (*R* ^2^_adjusted_), were used to identify the optimal model. Table S1 indicates that, in most cases, the first-order model and the Korsmeyer–Peppas model show comparable values for AIC and *R* ^2^_adjusted_. The first-order model highlights the impact of alginate concentration on decreasing the first-order release rate constant (see Figure 3C), suggesting that fluorescein release is concentration-dependent, with the release rate proportional to the remaining amount of dye within the porous cryogel matrix.^34^ Complementarily, the Korsmeyer–Peppas model provides additional mechanistic insights, with the release exponent (*n*) values ranging from 0.362 to 0.430 for formulations containing ≤0.75% alginate, and increasing to 0.797 and 0.568 for formulations with 1% and 2% alginate, respectively. Thus, depending on alginate concentration, fluorescein release appears to follow either Fickian diffusion (*n* ≤ 0.45) or non-Fickian (anomalous) mechanisms (0.45 < *n* < 0.89), involving a combination of drug diffusion and polymer matrix swelling.^34,35^

We further evaluated fluorescein release from albumin cryogels containing varying amounts of Na_2_HPO_4_, maintaining a fixed alginate level of 2%. All gel formulations exhibited excellent buoyancy and did not sink in any of the triplicate experiments (Figure S1A). The release rate of fluorescein generally decreased with increasing Na_2_HPO_4_ concentrations (Figure S1B). However, an anomalous release pattern was observed for the gel containing 480 mM Na_2_HPO_4_, which tended to rapidly disintegrate at the early stage, resulting in a significant increase in fluorescein release. This phenomenon might be attributed to the swelling of alginate at high Na_2_HPO_4_ levels, thereby facilitating gel disintegration upon wetting (Figure S1A). Interestingly, this facilitated disintegration did not enhance the cumulative fluorescein release at 24 h. Previously, we have shown that high phosphate concentrations yielded gels with a more hydrophobic and less porous gel matrix,^29,30^ which might account for the diminished overall release. Model fitting of the experimental data indicated that the first-order model was the most suitable for all formulations (Table S2), with the fitted first-order rate constant compared in Figure S1C.

Several formulation and experimental parameters were also investigated for their effect on fluorescein release. Figure S2 shows that the release rate of fluorescein only slightly decreased when albumin content in the preparation was increased from 5% to 10%. Altering the loading content of fluorescein did not significantly modify the release profile (Figure S3). Additionally, the difference in gelation protocol (room temperature for 24 h vs 50 °C for 1 h) did not affect the release profile (Figure S4). Finally, the release profile at 37 °C was similar to that at ambient temperature, with a predictable, marginal increase in release extent due to temperature (Figure S5). Therefore, for throughput considerations, all release studies were conducted at ambient temperature.

### 3.3 Controlled Release of Various Payloads

Based on the degradability test and the fluorescein loading and release study, the optimal cryogel formulation was identified as the one prepared from 5% w/v albumin, 120 mM MPTMS, 240 mM Na_2_HPO_4_, and 2% w/v alginate. Consequently, the general applicability of the proposed formulation as a controlled-release system was demonstrated in subsequent drug loading and release experiments with various payloads, including methylene blue, doxorubicin, rhodamine 6G, and mitoxantrone. Although the maximal extent of release varied among different payloads, Figures 4–7 reveal striking similarities in their release profiles. Regardless of the payload, these profiles were best described by the first-order model (Table S3). For the control formulation (without alginate), the fitted first-order release rate constant (*k*_1_) generally ranged from 1.0 to 1.5 h^–1^, whereas the rate constants obtained from the alginate groups consistently clustered around 0.5 h^–1^. Overall, we demonstrate that the optimal cryogel formulation exhibited consistent floating capability and reduced the release rate of various model payloads by 2–3-fold (Figures 4–7).

**Figure 4:**
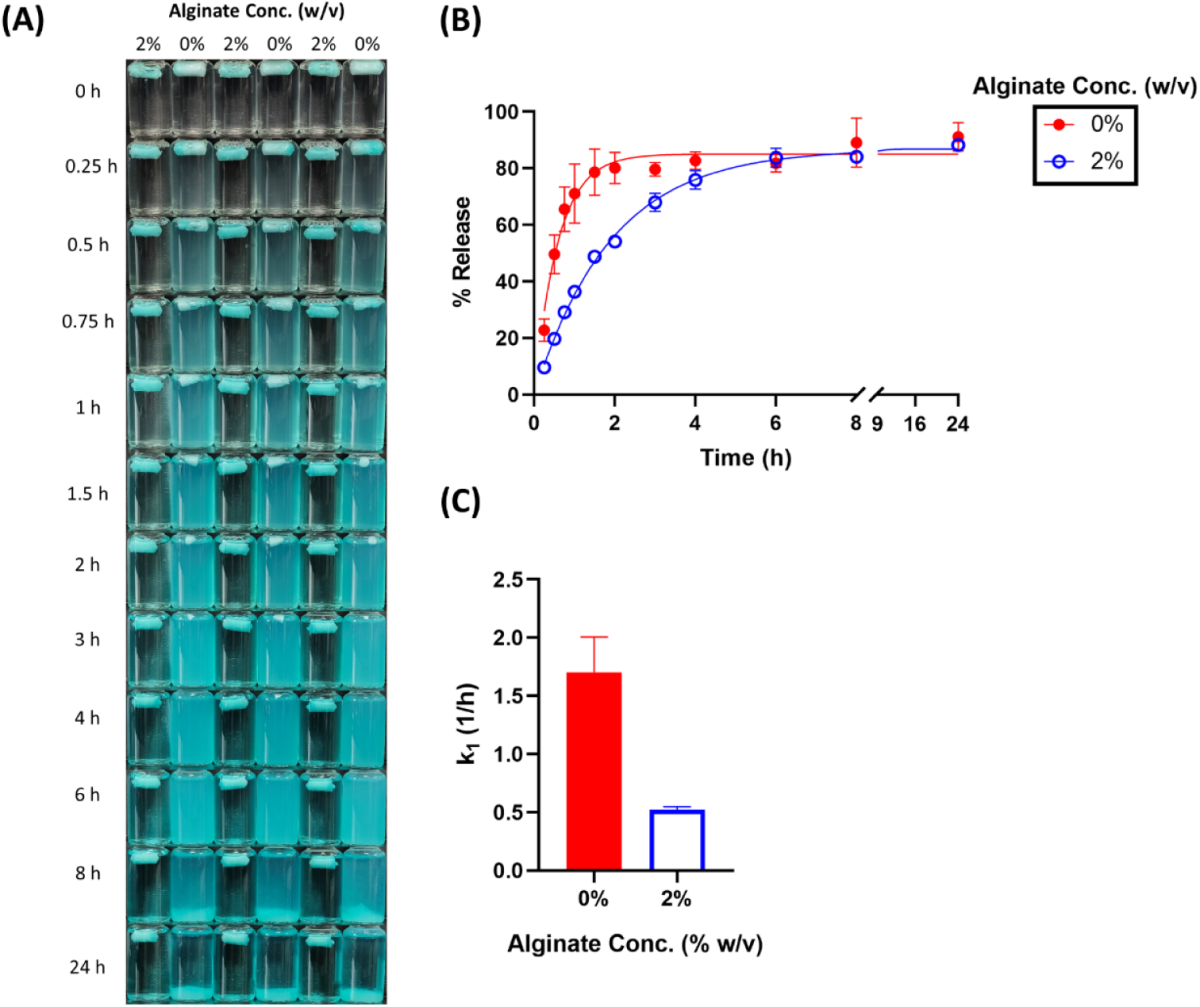
Effect of alginate concentration on the release of methylene blue in SGF. (A) Visual appearance of cryogels over time. (B) Cumulative release profiles with observed values (dots) and model fits (lines). (C) First-order release rate constants (*k*_1_) derived from model fitting.

**5:**
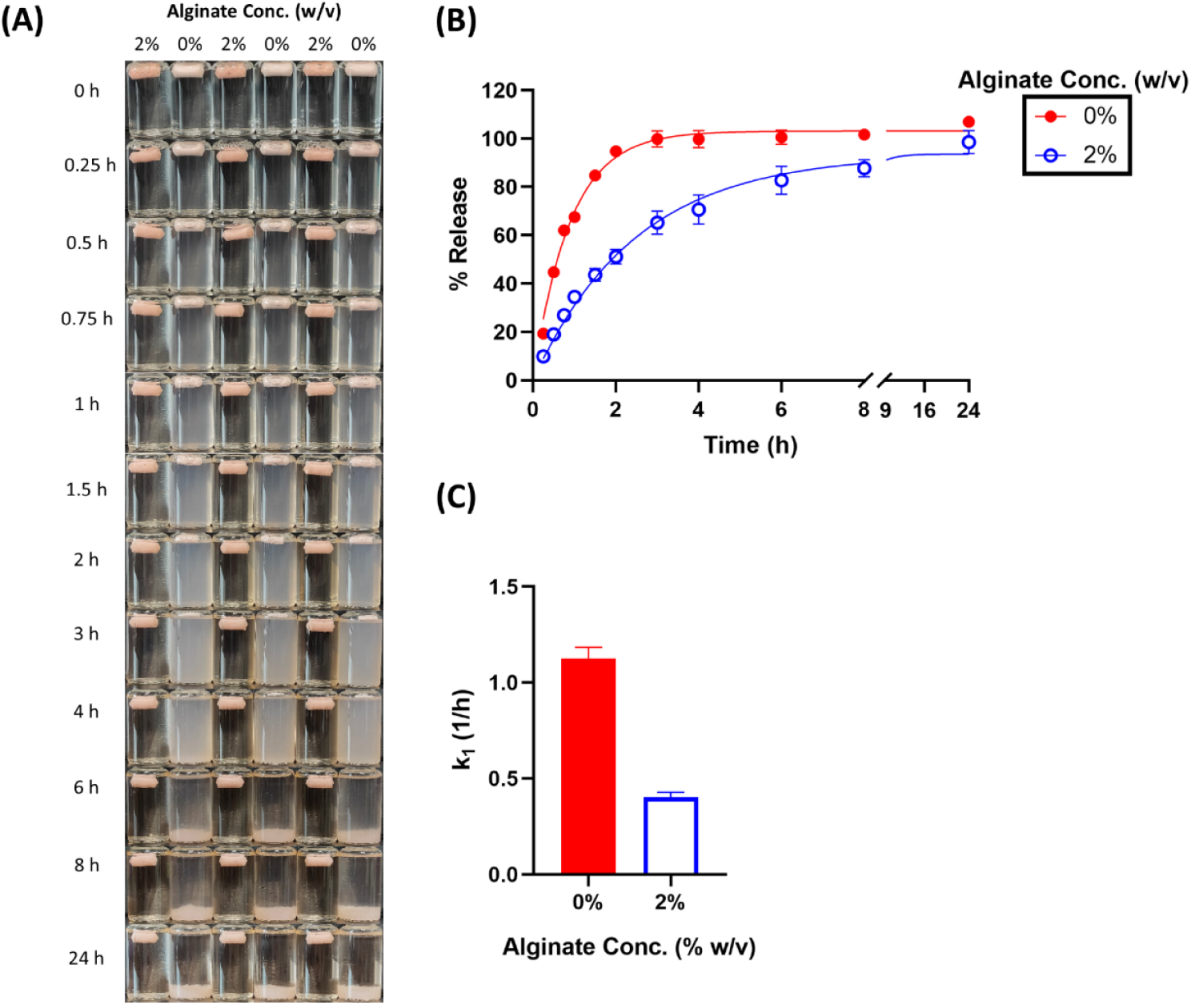
Effect of alginate concentration on the release of doxorubicin in SGF. (A) Visual appearance of cryogels over time. (B) Cumulative release profiles with observed values (dots) and model fits (lines). (C) First-order release rate constants (*k*_1_) derived from model fitting.

**6:**
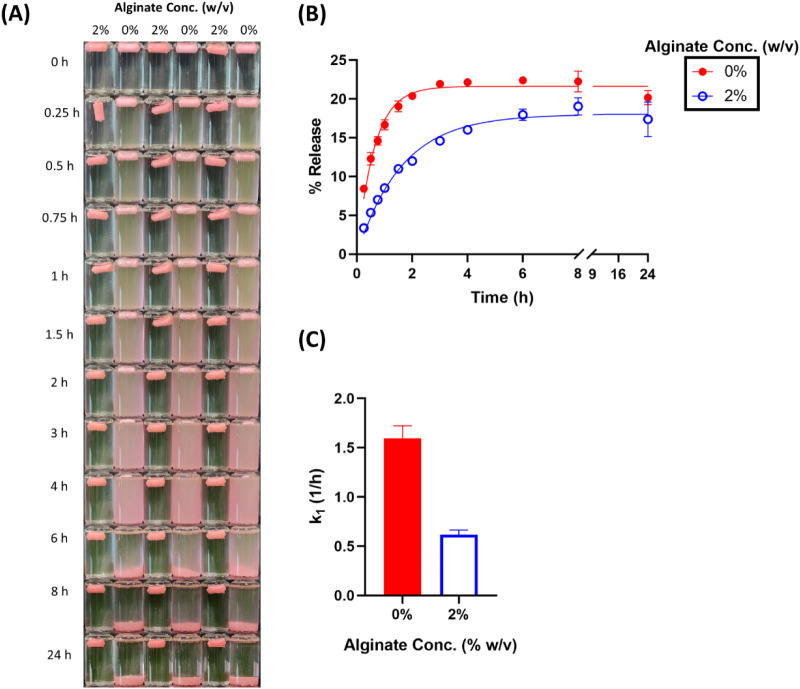
Effect of alginate concentration on the release of rhodamine 6G in SGF. (A) Visual appearance of cryogels over time. (B) Cumulative release profiles with observed values (dots) and model fits (lines). (C) First-order release rate constants (*k*_1_) derived from model fitting.

**Figure 7:**
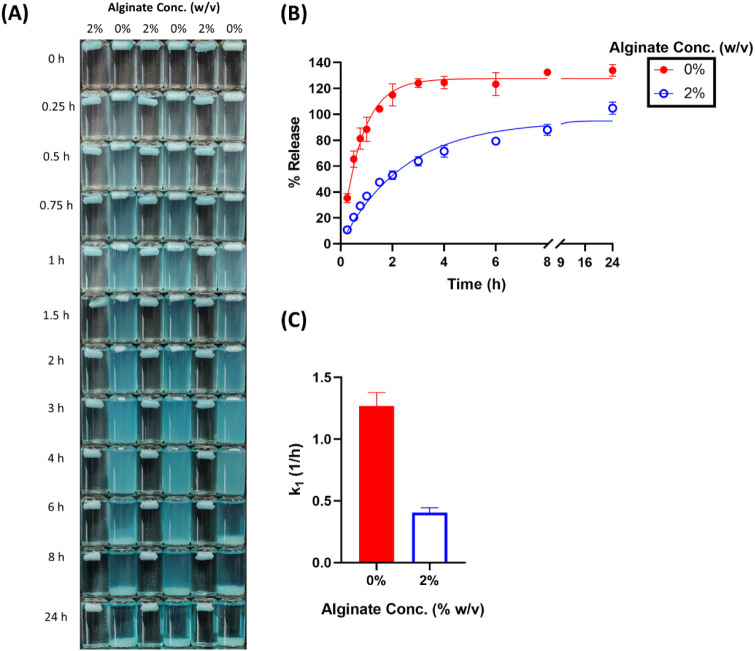
Effect of alginate concentration on the release of mitoxantrone in SGF. (A) Visual appearance of cryogels over time. (B) Cumulative release profiles with observed values (dots) and model fits (lines). (C) First-order release rate constants (*k*_1_) derived from model fitting. Release values >100% in the control group likely reflect fluorescence interference from dissolved albumin.

Taken together (Sections 3.2 and 3.3), the drug release mechanism of the cryogel system can be further understood as governed by both diffusion-controlled and matrix-controlled factors. The diffusion-controlled component primarily depends on the hydrophobicity and aqueous solubility of the payload molecules, as demonstrated by our findings,^29,30^ which showed that maximum release inversely correlated with model drug hydrophobicity. The matrix-controlled release behavior is attributable to the reduced swelling and increased hydrophobicity imparted by phosphate,^29,30^ as well as the partial resistance of the protein–siloxane–alginate network to enzymatic digestion (this study). While we did not directly quantify specific molecular interactions (e.g., ionic bonding or hydrogen bonding), the more uniform release rate constants observed among diverse model payloads in simulated gastric fluid suggest that the alginate component plays a key role in stabilizing the matrix and mitigating the influence of payload physicochemical properties. Future studies will investigate these specific binding interactions in more detail to further elucidate the retention mechanisms.

### 3.4 pH-Responsive Property

Figure S6 shows that the structure of cryogels containing 1–4% sodium alginate remained intact and stably floating in SGF over 24 h. In contrast, the gels rapidly disintegrated (within 10 min) upon placing in water or simulated intestinal fluid (SIF), especially for gels prepared with higher alginate concentrations (e.g., 2% and above). The pH responsiveness was further revealed in a subsequent experiment where the gels were placed in both SGF and SIF sequentially. Upon SGF to SIF transition, the gel containing 2% alginate instantly disintegrated in SIF (Figure 8). This property could be explained by the swelling and water-wicking capability of alginate in an environment where the pH is above the p*K*_a_ value (i.e., 3.5–4.6) of alginic acid.^36^

**Figure 8:**
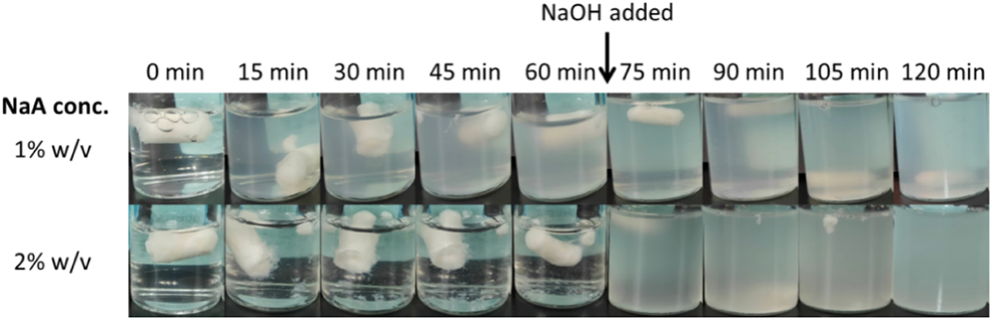
pH-responsive behavior of the cryogel. Cryogel monoliths were placed in 10 mL SGF. After 60 min, the pH was adjusted to 6.8 by adding a KH_2_PO_4_/NaOH solution. Structural changes were monitored before and after pH adjustment.

### 3.5 SEM Images

The SEM images (Figure 9) illustrate the matrix structure of cryogels prepared with varying alginate concentrations. Without alginate, the cryogel exhibits macroporous inner structure, with pore sizes measuring several tenths of a micrometer, separated by granular yet intact and continuous walls. The introduction of alginate significantly alters the porous architecture. At 0.5% w/v alginate, the cryogel shows a more compact and dense cross-sectional structure, characterized by less distinct pores with narrower sizes. Additionally, the interpore walls appear perforated, fragmented, and filled with fibrous entanglements. As the alginate concentration increases to 2%, the fibrous structures become more pronounced, resulting in a less porous and shallower inner structure. Based on these SEM images, it can be reasonably inferred that the enhanced resistance of the cryogel to gastric fluid by alginate is not solely due to alginate’s intrinsic ability to inhibit pepsin. The densification of the cryogel structure and reduction in pore size likely contribute to the mechanical slowing of gastric fluid penetration.

**Figure 9:**
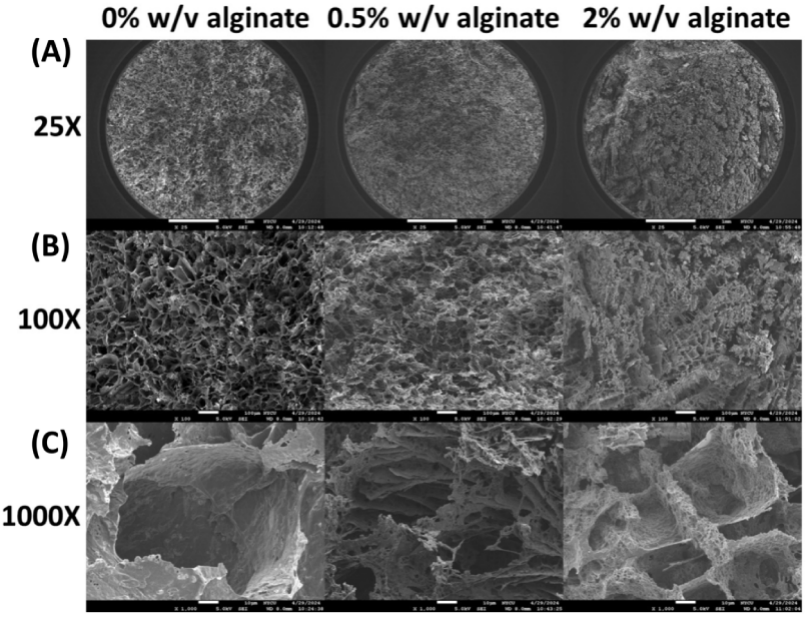
SEM images of albumin cryogels with increasing alginate concentrations. (A) 25×, (B) 100×, and (C) 1000× magnification. Images show changes in porosity and fibrous density as alginate concentration increases.

### 3.6 Significance and Perspectives

In this study, we explore the potential of albumin–organosilane composite cryogels as gastroretentive drug delivery systems (GRDDS). These cryogels are synthesized using a straightforward, room-temperature gelation method, in which albumin solutions are cross-linked in the presence of 3-mercaptopropyltrimethoxysilane (MPTMS). The aqueous precursor contains only albumin, MPTMS, and dibasic sodium phosphate, and gelation proceeds without the need for strong acids, bases, or other catalysts. While gelation typically takes 1 day at room temperature, it can be significantly accelerated by elevating the temperature, reducing the time to 1 h at 50 °C. Following lyophilization, a lightweight cryogel monolith is obtained. We demonstrated the cryogel’s excellent buoyancy in water—an essential feature for GRDDS—though its protein component was found to degrade rapidly in gastric fluid. The present study, therefore, focuses on optimizing this system to balance floatability with controlled degradability.

This study introduces a novel gastroretentive drug delivery platform based on albumin-organosilica-alginate composite cryogels, offering a biocompatible and tunable alternative to existing polymer-based systems. The protein-based cryogels demonstrate excellent floatability and can be engineered for controlled degradation in gastric conditions, highlighting their potential for delivering drugs with narrow absorption windows or those unstable at intestinal pH. Beyond traditional drug delivery, the platform’s structural versatility and bioderived composition may open possibilities for emerging applications, such as integration with bioelectronic sensors for real-time gastric diagnostics (e.g., for gastric ulcers, *H. pylori* infection, or gastric emptying disorders) or as carriers for responsive, stomach-resident therapeutics (e.g., live probiotics.)^5–11,37,38^ These findings highlight the broader potential of floating protein matrices in both therapeutic and diagnostic innovations within the gastrointestinal tract.

Notably, the use of protein-based materials in oral delivery is traditionally considered unfavorable due to their rapid degradation in the gastric environment. This study challenges that paradigm by demonstrating that albumin, when integrated into an organosilane-cross-linked cryogel matrix reinforced by incorporated alginate, can retain structural integrity long enough to serve as a functional gastroretentive platform. By leveraging mild processing conditions and a hybrid organic–inorganic architecture incorporating alginate as a pepsin inhibitor, we demonstrate that the intrinsic instability of proteins under acidic conditions can be effectively mitigated. This not only validates the feasibility of protein-based floating systems but also highlights the broader potential of biosourced, biocompatible materials for oral drug delivery applications previously thought unsuitable for proteins.

Alginate-based gastroretentive drug delivery systems have been extensively studied and categorized into multiple platform strategies, including low-density systems,^37,39,40^ mucoadhesive delivery systems,^38,41^ hydrogels,^42^ and others.^21^ While these approaches often rely on gel-forming capabilities in combination with auxiliary polysaccharides or synthetic polymers, to the best of our knowledge, our system is the first to employ a low-density protein–organosilica composite cryogel as the primary structural framework, with incorporated alginate serving as a functional modulator of enzymatic degradation. This combination offers a distinctive advantage: the ability to tune the cryogel’s degradability in gastric conditions and achieve controlled release of loaded drugs. Unlike previously reported floating or alginate-based systems, this strategy demonstrates that integrating albumin with organosilane cross-linking and alginate incorporation can overcome the inherent susceptibility of proteins to gastric enzyme-mediated breakdown, thereby establishing a novel, biocompatible gastroretentive platform.

While these findings are promising, it is important to note that the performance of the cryogels was evaluated using in vitro models based on simulated gastric and intestinal fluids. These models do not fully replicate the complex and dynamic physiological environment of the gastrointestinal tract, including factors such as gastric motility, mucus interaction, enzymatic variability, and mechanical forces. Therefore, future in vivo studies will be essential to confirm the cryogels’ gastroretentive behavior and drug release kinetics under physiological conditions. In addition, although the present study did not include in vitro biocompatibility evaluations, our previous reports have demonstrated that organosilica-based materials prepared with the same organosilane precursor (MPTMS) are generally biocompatible with normal mammalian cells and erythrocytes, as well as in rats.^30,43–45^ Nevertheless, dedicated studies assessing in vitro and in vivo biocompatibility under oral administration conditions will be essential to fully establish the safety of this platform.

## 4 Conclusions

This study presents a novel albumin-based cryogel platform for gastroretentive drug delivery, combining sustained buoyancy, tunable resistance to gastric degradation, and controlled drug release. Through thiol-organosilane-mediated gelation and lyophilization, structurally robust cryogels were fabricated under mild, biocompatible conditions. The incorporation of Na_2_HPO_4_ and alginate enhanced the cryogel’s gastric stability and enabled pH-responsive behavior, while SEM analysis revealed a denser microstructure correlating with slowed release of multiple model compounds. By overcoming the inherent instability of proteins in acidic environments, this work establishes the feasibility of using biosourced protein matrices for oral delivery. Beyond GRDDS, the platform’s modularity and structural versatility suggest potential future applications in gastric-resident diagnostics, probiotic delivery, or bioresponsive therapeutic systems.

Underlying these advances is the strategic selection of albumin as the foundational material. Albumin is a biocompatible, biodegradable plasma protein widely recognized for its ability to bind and stabilize diverse therapeutic agents. Its renewable sourcing and compatibility with mild processing conditions make it an attractive material for developing sustainable, eco-friendly drug delivery systems. These attributes support the use of albumin-based cryogels as versatile and environmentally conscious platforms for biomedical applications.

## Supplementary Material



## Notes

Figure S1: effect of Na_2_HPO_4_ concentration on fluorescein release in SGF; Figure S2: effect of albumin concentration on fluorescein release in SGF; Figure S3: effect of loaded fluorescein concentration on sodium fluorescein (NaFl) release in SGF; Figure S4: effect of gelation condition on fluorescein release in SGF; Figure S5: comparison of fluorescein release in simulated gastric fluid (SGF) at two temperatures; Figure S6: effect of sodium alginate (NaA) concentrations on pH-responsive disintegration of cryogels; Table S1: model discrimination based on goodness-of-fit indicators for fluorescein release from cryogels with varying alginate concentrations; Table S2: model discrimination based on goodness-of-fit indicators for fluorescein release from cryogels with varying Na_2_HPO_4_ concentrations; Table S3: model discrimination for drug release profiles from cryogels with and without alginate. (PDF)
