# Supplementary material for: Albumin-Based Cryogels as Floating Platforms for Gastroretentive Drug Delivery Applications
Source: ACS Omega. 2025 Aug 13;10(33):37639–49. doi: 10.1021/acsomega.5c04153 (PMC12392197; doi:10.1021/acsomega.5c04153)
Supplement: Supplementary file 1 [file ao5c04153_si_001.pdf]

Supplementary Materials

Albumin-Based Cryogels as Floating Platforms for Gastro-Retentive Drug Delivery Applications

Wei-Chin Hsu and Teh-Min Hu*

Department of Pharmacy, College of Pharmaceutical Sciences, National Yang Ming Chiao Tung University, Taipei 112304, Taiwan

*Corresponding author: T.-M. Hu, tehmin@nycu.edu.tw

Keywords: Albumin, Cryogels, Alginate, Gastro-retentive drug delivery systems

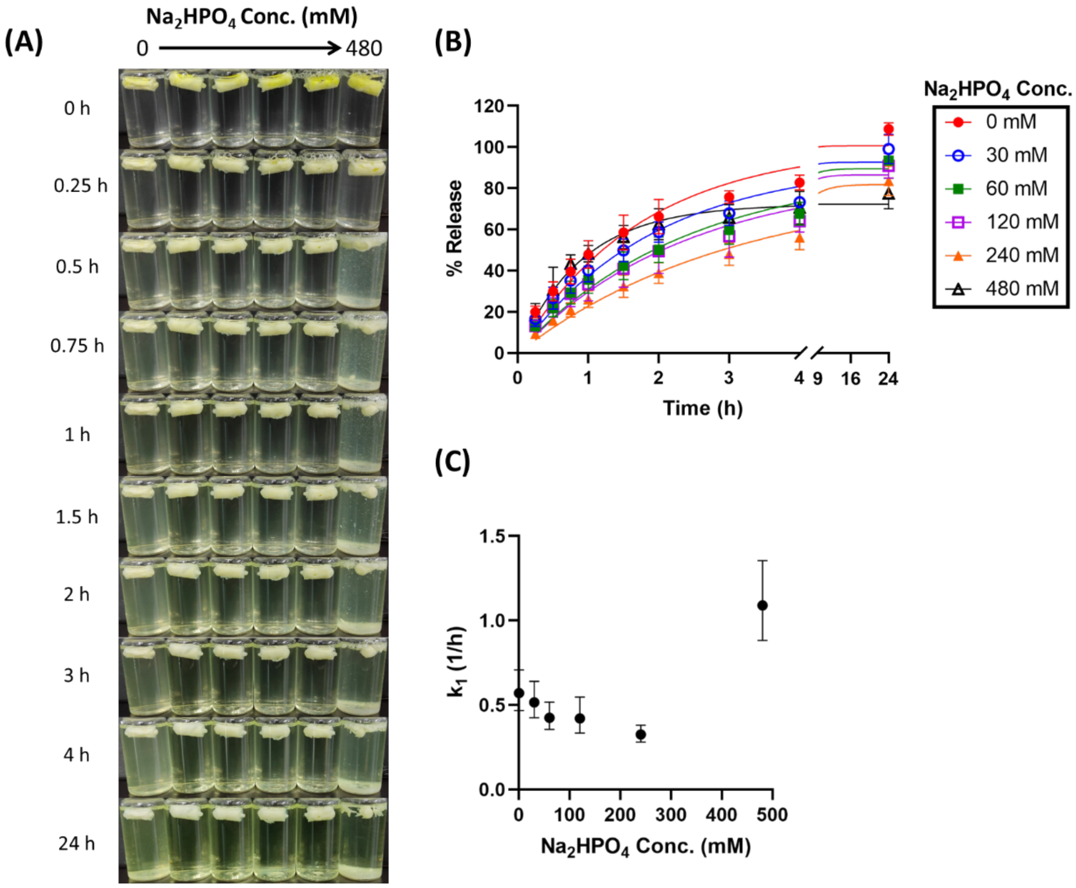

Figure S1. Effect of Na_2HPO_4 concentration on fluorescein release in SGF. (A) Cryogel appearance over time. (B) Cumulative release profiles (dots) and first-order model fits (lines). (C) Corresponding release rate constants (k_1).

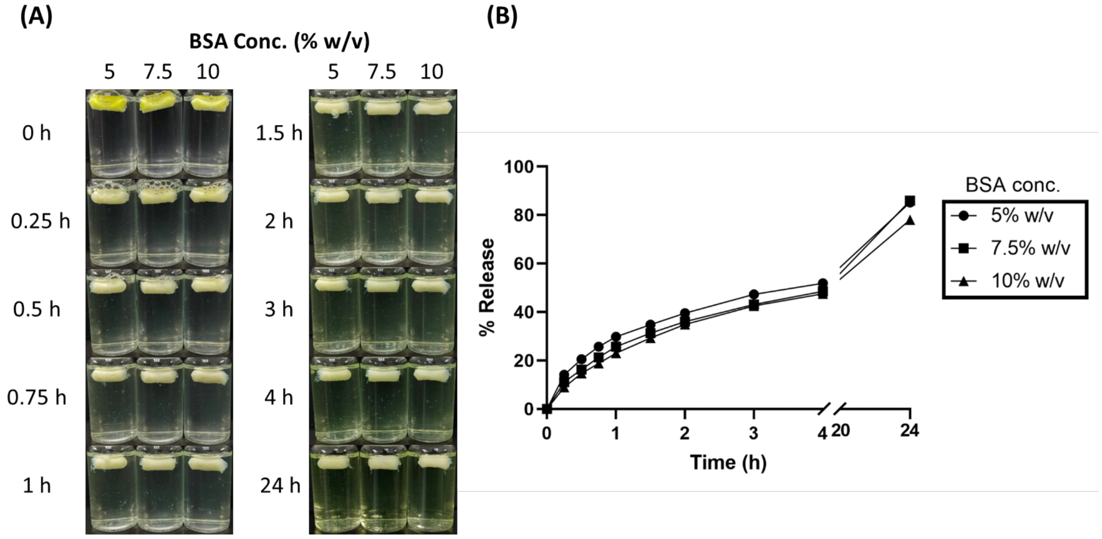

Figure S2. Effect of albumin concentration on fluorescein release in SGF. (A) Cryogel appearance over time. (B) Cumulative release profiles.

Cryogels were prepared with varying albumin (BSA) concentrations (5%, 7.5%, or 10% w/v) while maintaining fixed compositions of 120 mM MPTMS, 240 mM Na_2HPO_4, 2% w/v alginate, and 100 µM fluorescein. Gelation was conducted at ambient temperature for 24 hours.

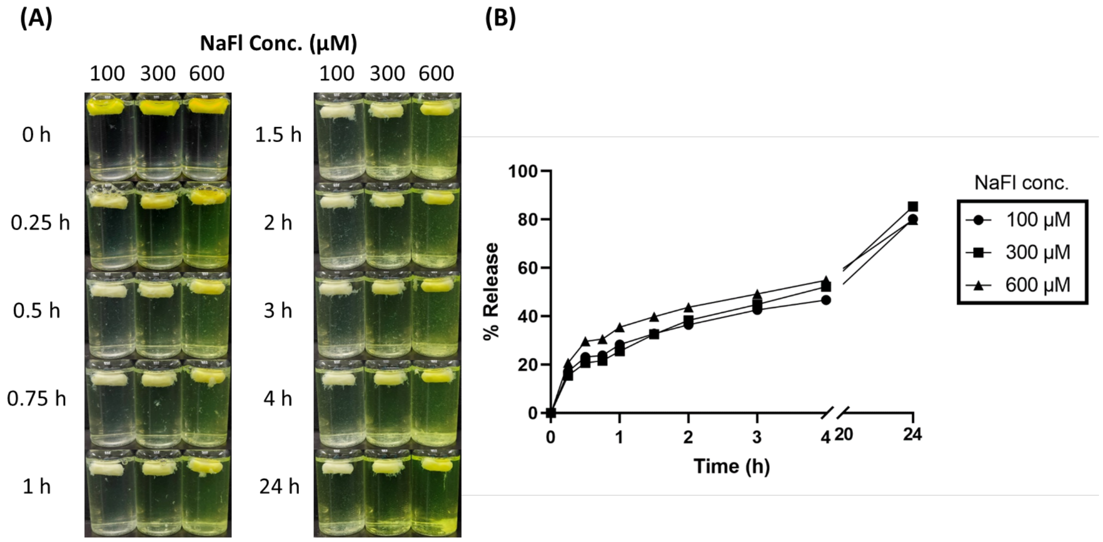

Figure S3. Effect of loaded fluorescein concentration on sodium fluorescein (NaFl) release in SGF. (A) Cryogel appearance over time. (B) Cumulative release profiles.

Cryogels were prepared with varying concentrations of sodium fluorescein loaded (100, 300, or 600 μM) while maintaining fixed compositions of 5% BSA, 120 mM MPTMS, 240 mM Na_2HPO_4, 2% w/v alginate. Gelation was conducted at ambient temperature for 24 hours.

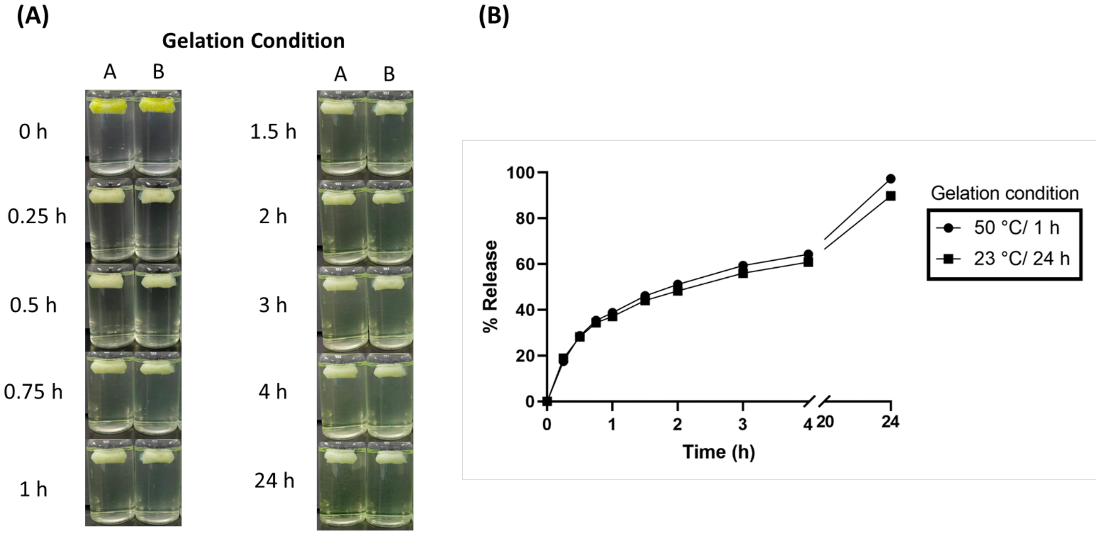

Figure S4. Effect of gelation condition on fluorescein release in SGF. (A) Cryogel appearance over time. (B) Cumulative release profiles.

Cryogels were prepared with varying gelation condition (50°C/1h vs. ambient temperature/24 h) while maintaining fixed compositions of 5% BSA, 120 mM MPTMS, 240 mM Na_2HPO_4, 2% w/v alginate.

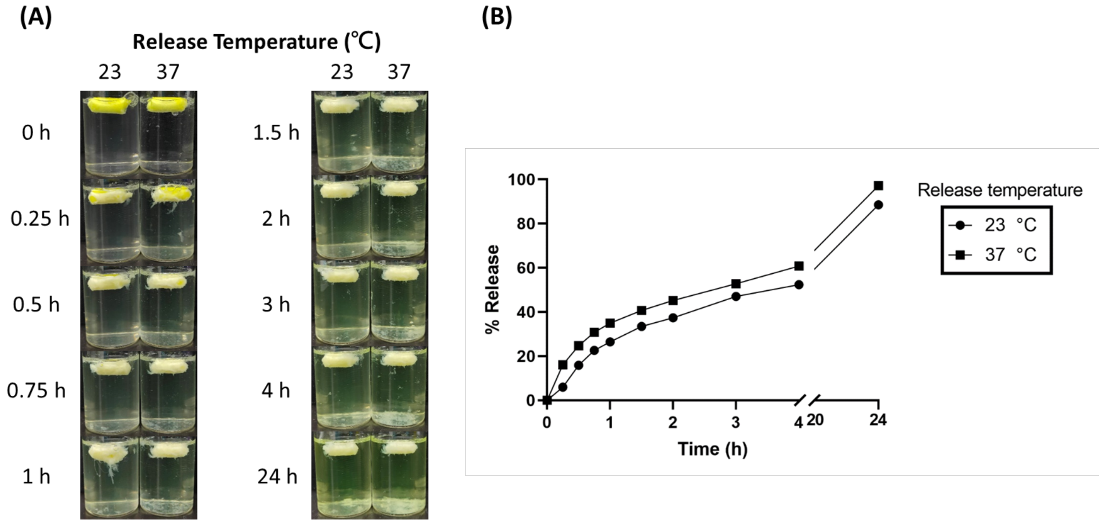

Figure S5. Comparison of fluorescein release in simulated gastric fluid (SGF) at two temperatures. (A) Cryogel appearance over time. (B) Cumulative release profiles.

Cryogels were prepared with 5% w/v BSA, 120 mM MPTMS, 240 mM Na_2HPO_4, and 2% w/v alginate. Gelation was carried out at ambient temperature for 24 hours.

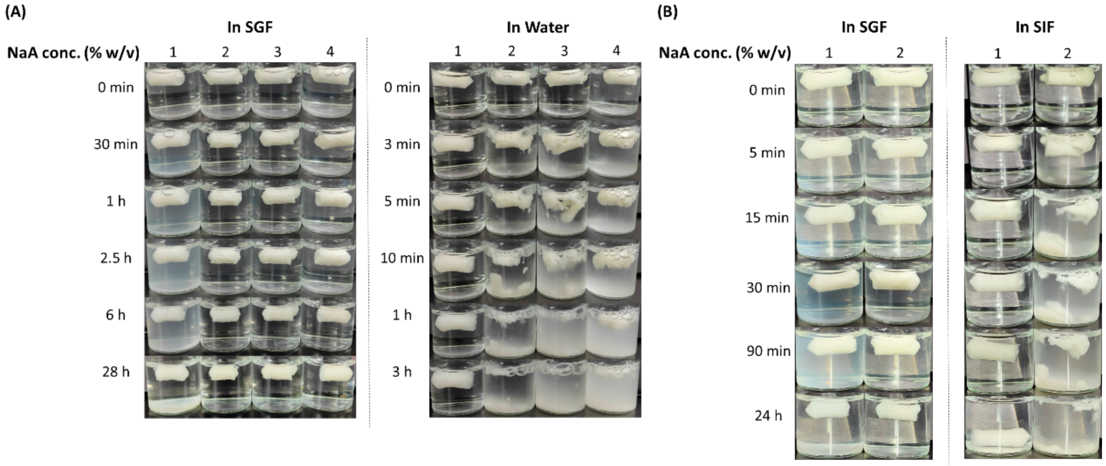

Figure S6. Effect of sodium alginate (NaA) concentrations on pH-responsive disintegration of cryogels. (A) Cryogels immersed in SGF or water. (B) Cryogels immersed sequentially in SGF and simulated intestinal fluid (SIF). Structural integrity changes reflect pH-triggered disintegration.

Table S1. Model discrimination based on goodness-of-fit indicators for fluorescein release from cryogels with varying alginate concentrations.

	0% w/v		0.25% w/v		0.5% w/v		0.75% w/v		1% w/v		2% w/v	
	AIC	R^2 adjusted	AIC	R^2 adjusted	AIC	R^2 adjusted	AIC	R^2 adjusted	AIC	R^2 adjusted	AIC	R^2 adjusted
Zero-order	99.0	-7.89	88.9	-4.10	86.0	-2.46	82.1	-1.27	76.4	0.159	73.4	0.0417
First-order	**60.1**	**0.891**	57.2	0.861	57.6	0.865	56.4	0.881	**27.3**	**0.997**	43.2	0.970
Higuchi	91.8	-3.02	77.9	-0.503	73.5	0.141	66.9	0.579	59.5	0.871	51.2	0.919
Hixson-Crowell	93.7	-4.00	79.1	-0.727	74.8	-0.0009	68.9	0.476	59.3	0.874	64.6	0.642
Korsmeyer-Peppas	77.3	0.266	**56.1**	**0.877**	**53.2**	**0.917**	**46.6**	**0.960**	59.0	0.889	**42.7**	**0.971**

The release data were analyzed by fitting to five kinetic models using DDsolver. AIC: Akaike Information Criterion. $R^2_{adjusted}$: Adjusted coefficient of determination. Boldface highlights the model with the best overall fit (lowest AIC and highest $R^2_{adjusted}$) for each alginate concentration.

Table S2. Model discrimination based on goodness-of-fit indicators for fluorescein release from cryogels with varying Na_2HPO_4 concentrations.

	0 mM		30 mM		60 mM		120 mM		240 mM		480 mM	
	AIC	$R^2_{adjusted}$	AIC	$R^2_{adjusted}$	AIC	$R^2_{adjusted}$	AIC	$R^2_{adjusted}$	AIC	$R^2_{adjusted}$	AIC	$R^2_{adjusted}$
Zero-order	89.9	-1.79	87.4	-1.51	84.8	-0.978	84.0	-0.987	80.0	-0.354	89.3	-4.47
**First-order**	**54.7**	**0.949**	**52.7**	**0.952**	**49.2**	**0.966**	**50.9**	**0.955**	**41.9**	**0.982**	**40.6**	**0.978**
Higuchi	77.1	0.329	74.1	0.433	70.2	0.608	69.2	0.618	62.9	0.796	79.6	-0.861
Hixson-Crowell	79.3	0.141	75.3	0.351	70.5	0.595	69.7	0.597	63.2	0.790	80.4	-1.03
Korsmeyer-Peppas	62.6	0.877	60.3	0.888	58.7	0.900	56.5	0.915	54.9	0.923	63.7	0.710

The release data were analyzed by fitting to five kinetic models using DDsolver. AIC: Akaike Information Criterion. $R^2_{adjusted}$: Adjusted coefficient of determination. Boldface highlights the model with the best overall fit (lowest AIC and highest $R^2_{adjusted}$) for each Na_2HPO_4 concentration.

Table S3. Model discrimination for drug release profiles from cryogels with and without alginate.

	Methylene blue				Doxorubicin				Rhodamine 6G				Mitoxantrone			
	0% w/v		2% w/v		0% w/v		2% w/v		0% w/v		2% w/v		0% w/v		2% w/v	
	AIC	$R^2_{adjusted}$	AIC	$R^2_{adjusted}$	AIC	$R^2_{adjusted}$	AIC	$R^2_{adjusted}$	AIC	$R^2_{adjusted}$	AIC	$R^2_{adjusted}$	AIC	$R^2_{adjusted}$	AIC	$R^2_{adjusted}$
Zero-order	117	-7.48	109	-1.21	119	-4.16	107	-0.673	87.1	-9.50	76.6	-2.00	124	-5.70	107	-0.661
**First-order**	**60.0**	**0.955**	**34.3**	**0.998**	**52.8**	**0.988**	**51.9**	**0.990**	**24.7**	**0.967**	**19.8**	**0.984**	**60.0**	**0.982**	**65.5**	**0.966**
Higuchi	105	-1.93	92.9	0.498	106	-0.622	88.2	0.711	76.0	-2.82	61.6	0.232	111	-1.11	86.6	0.749
Hixson-Crowell	107	-2.59	90.7	0.588	109	-1.14	85.4	0.776	86.4	-8.86	75.9	-1.81	117	-2.55	86.0	0.761
Korsmeyer-Peppas	83.3	0.624	84.3	0.787	89.8	0.663	80.4	0.868	50.1	0.751	52.9	0.566	90.1	0.720	76.4	0.908

Drug release data for methylene blue, doxorubicin, rhodamine 6G, and mitoxantrone were fitted to standard kinetic models. Model selection was based on AIC and $R^2_{adjusted}$. Boldface denotes the best-fitting model for each drug and formulation.
